# Microbiome Datasets Are Compositional: And This Is Not Optional

**DOI:** 10.3389/fmicb.2017.02224

**Published:** 2017-11-15

**Authors:** Gregory B. Gloor, Jean M. Macklaim, Vera Pawlowsky-Glahn, Juan J. Egozcue

**Affiliations:** ^1^Department of Biochemistry, University of Western Ontario, London, ON, Canada; ^2^Departments of Computer Science, Applied Mathematics, and Statistics, Universitat de Girona, Girona, Spain; ^3^Department of Applied Mathematics, Universitat Politècnica de Catalunya, Barcelona, Spain

**Keywords:** microbiota, compositional data, high-throughput sequencing, correlation, Bayesian estimation, count normalization, relative abundance

## Abstract

Datasets collected by high-throughput sequencing (HTS) of 16S rRNA gene amplimers, metagenomes or metatranscriptomes are commonplace and being used to study human disease states, ecological differences between sites, and the built environment. There is increasing awareness that microbiome datasets generated by HTS are compositional because they have an arbitrary total imposed by the instrument. However, many investigators are either unaware of this or assume specific properties of the compositional data. The purpose of this review is to alert investigators to the dangers inherent in ignoring the compositional nature of the data, and point out that HTS datasets derived from microbiome studies can and should be treated as compositions at all stages of analysis. We briefly introduce compositional data, illustrate the pathologies that occur when compositional data are analyzed inappropriately, and finally give guidance and point to resources and examples for the analysis of microbiome datasets using compositional data analysis.

## 1. Introduction

The collection and analysis of microbiome datasets presents many challenges in the study design, sample collection, storage, and sequencing phases, and these have been well reviewed (Robinson et al., 2016). Many methods for the analysis of microbiome datasets assume that sequencing data are equivalent to ecological data where the counts of reads assigned to organisms are often normalized to a constant area or volume. Methods applied include count-based strategies such as Bray-Curtis dissimilarity, zero-inflated Gaussian models and negative binomial models (McMurdie and Holmes, 2014; Weiss et al., 2017).

In an ecological study it is possible for many different species to co-exist, and their absolute abundance may be important. For example, in an area containing only tigers, it is important to know if the population size is sufficient to maintain needed genetic diversity for long-term survival (Shaffer, 1981). However, the abundance of one species may not influence the abundance of another; the area may contain both tigers and ladybugs, and the migration of several ladybugs into the area would not be expected to affect the number of tigers.

**Figure 1:**
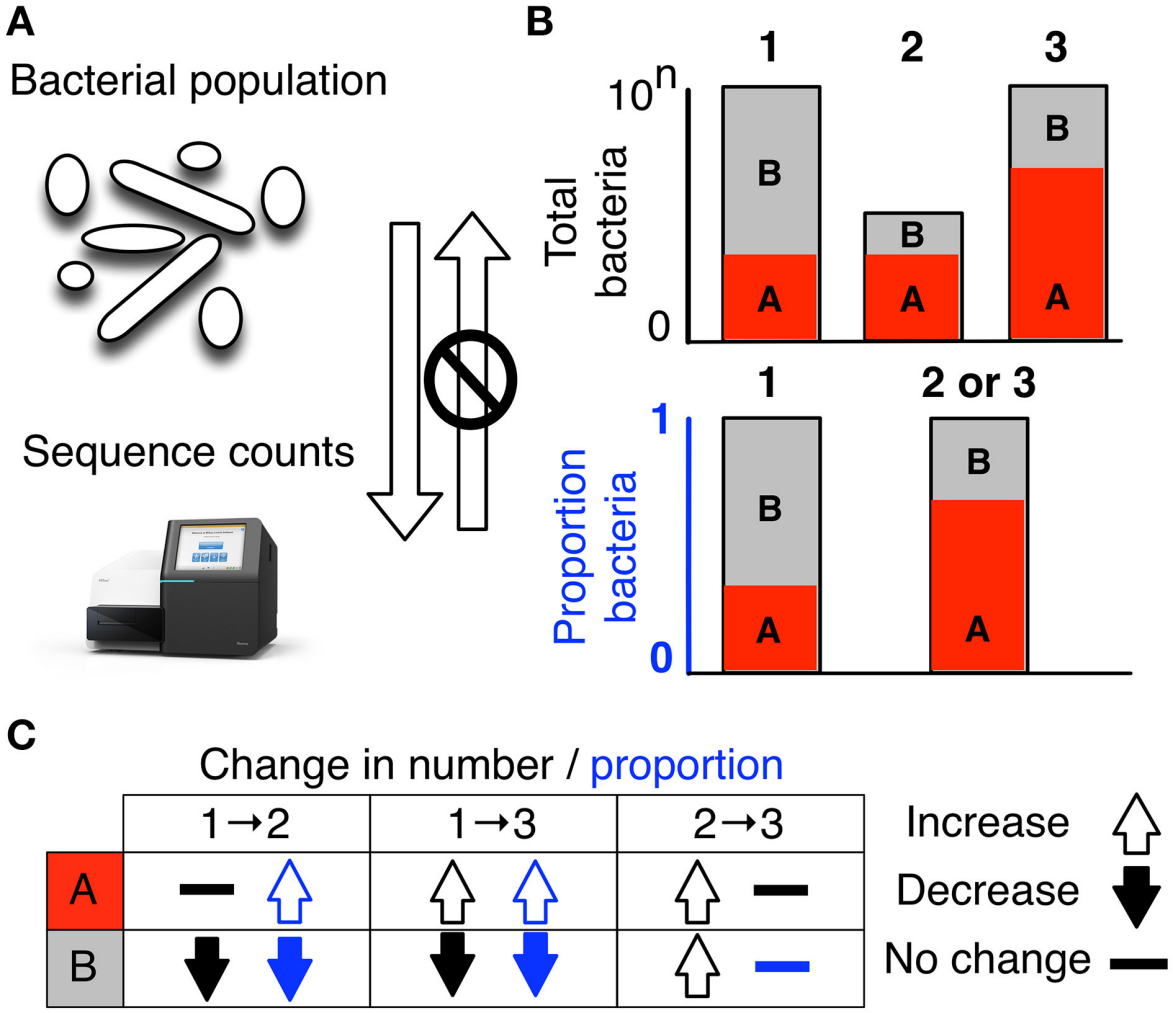
High-throughput sequencing data are compositional. **(A)** illustrates that the data observed after sequencing a set of nucleic acids from a bacterial population cannot inform on the absolute abundance of molecules. The number of counts in a high throughput sequencing (HTS) dataset reflect the proportion of counts per feature (OTU, gene, etc.) per sample, multiplied by the sequencing depth. Therefore, only the relative abundances are available. The bar plots in **(B)** show the difference between the count of molecules and the proportion of molecules for two features, A (red) and B (gray) in three samples. The top bar graphs show the total counts for three samples, and the height of the color illustrates the total count of the feature. When the three samples are sequenced we lose the absolute count information and only have relative abundances, proportions, or “normalized counts” as shown in the bottom bar graph. Note that features A and B in samples 2 and 3 appear with the same relative abundances, even though the counts in the environment are different. The table below in **(C)** shows real and perceived changes for each sample if we transition from one sample to another.

The assumption of true independence can not hold in high-throughput sequencing (HTS) experiments because the sequencing instruments can deliver reads only up to the capacity of the instrument. Thus, it is proper to think of these instruments as containing a fixed number of slots which must be filled. Returning to our tiger and ladybug analogy, the migration of ladybugs into an area containing a fixed number of slots that are already filled must displace either tigers or ladybugs from the occupied slots. This analogy extents, without restriction, to any number of taxa, and to any fixed capacity instrument (Aitchison, 1986; Lovell et al., 2011; Friedman and Alm, 2012; Fernandes et al., 2013, 2014; Lovell et al., 2015; Mandal et al., 2015; Gloor et al., 2016a,b; Gloor and Reid, 2016; Tsilimigras and Fodor, 2016). Thus, the total read count observed in a HTS run is a fixed-size, random sample of the relative abundance of the molecules in the underlying ecosystem. Moreover, the count can not be related to the absolute number of molecules in the input sample as shown in Figure 1. This is implicitly acknowledged when microbiome datasets are converted to relative abundance values, or normalized counts, or are rarefied (McMurdie and Holmes, 2014; Weiss et al., 2017) prior to analysis. Thus the number of reads obtained is irrelevant, and contains only information on the precision of the estimate (Fernandes et al., 2013). Data that are naturally described as proportions or probabilities, or with a constant or irrelevant sum, are referred to as compositional data. Compositional data contains information about the relationships between the parts (Aitchison, 1986; Pawlowsky-Glahn et al., 2015).

Data about a microbiome collected by high throughput sequencing are often examined under the assumption that sequencing is, in some way, *counting the number of molecules associated with the bacteria in the population*, as illustrated by the top barplot in Figure 1B. We can see the difference between counts and compositions by comparing the data for the actual counts for three samples in the top barplot with their proportions in the bottom barplot. Note, that samples 2 and 3 in Figure 1B have the same proportional abundances even though they have different absolute counts prior to sequencing. The difference in apparent direction of change is shown in Figure 1C and we can observe that the relationship between absolute abundance in the environment and the relative abundance after sequencing is not predictable.

## 2. Problems with current methods of analysis

**Figure 2:**
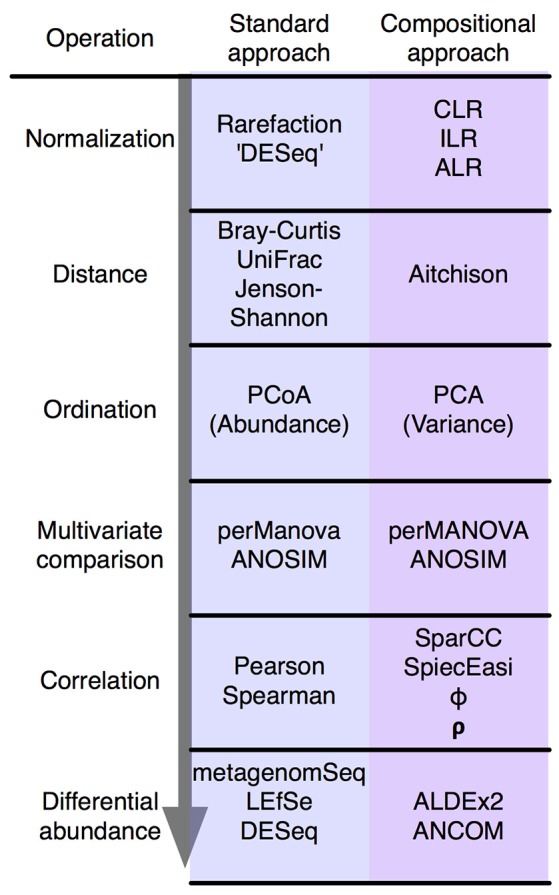
The standard microbiome analysis tool kit and the compositional replacements. A simplified standard microbiome computational workflow is illustrated. The initial normalization steps are not formally equivalent since compositional data are inherently “normalized”, and read count normalization is unnecessary. The other steps are functionally equivalent and substitute a compositionally appropriate approach for one that is not.

We will briefly outline the problems that arise when compositional data are examined using a non-compositional paradigm, stepping through the usual stages of analysis shown in Figure 2. All these issues have been extensively reviewed and debated in both the older and the more recent literature in fields as diverse as economics, geology and ecology. Thus, rather than present an exhaustive explanation of the problems, we will outline the major issue and cite a few useful resources.

It is very difficult to collect exactly the same number of sequence reads for each sample. This can be because of differences in platform (e.g., MiSeq vs. HiSeq) or because of technical difficulties in loading the same molar amounts of the sequencing libraries on the instrument, or because of random variation. The total number of counts observed (often referred to as read depth) is a major confounder for distance or dissimilarity calculations for multivariate ordinations derived from these distances (McMurdie and Holmes, 2014). Initial attempts in the microbiome field used “rarefaction” or subsampling of the read counts of each sample to a common read depth to attempt to correct this problem (Lozupone et al., 2011; Wong et al., 2016). The use of subsampling has been questioned since it results in a loss of information and precision (McMurdie and Holmes, 2014), and the practice of count normalization from the RNA-seq field has been advocated instead. There are a number of count normalization methods used and two, the trimmed mean of *M* values (TMM) (Robinson and Oshlack, 2010), and the median method (Anders and Huber, 2010) are similar to a log-ratio transformations, but are less suitable in highly asymmetrical or sparse datasets (Fernandes et al., 2013; Gloor et al., 2016a). These transformations are further undesirable since the number of counts observed by the instrument, by design, can not contain any information on the actual number of molecules in the environment, and because the investigator naturally interprets the results as counts instead of log-ratios.

One of the first analysis steps in a traditional analysis, following rarefaction or count normalization, is the calculation of a distance or dissimilarity (DD) matrix from the data that is used for downstream analyses such as ordination, and discrimination. Distances between features are non-linear when examined from a Euclidian perspective (Martín-Fernández et al., 1998; Aitchison et al., 2000) and many DD matrices are used that partially address this problem. As noted above the total number of reads in a sample is a strong confounding variable on all these methods, indicating that the composition of the sample is not the primary property being measured. However, apparently useful DD matrices can be generated after normalization. Three DD matrices dominate the literature; UniFrac (both the weighted and unweighted variants) (Lozupone et al., 2011), Bray-Curtis and Jensen-Shannon divergence, and while all have their uses, they do not account for the compositional nature of the data. It should be noted that the weighted UniFrac distance approach captures important phylogenetic information, and a recent compositional replacement has been developed (Silverman et al., 2017).

The major uses for the DD matrices are ordination and clustering. Here, the shortcomings of these DD methods become apparent. In addition to being sensitive to the total read depth of a sample, DD methods largely discriminate between samples based on the most relatively abundant features in the samples, not on the features that are necessarily the most variable between samples (Gorvitovskaia et al., 2016; Wong et al., 2016). This can lead to the location of samples in an ordination changing dramatically when different features are included or excluded from the dataset, and to a lack of sensitivity in identifying outlier samples (Wong et al., 2016).

Severe problems with correlation in compositional data were first noted at the dawn of statistical practice by Pearson (1897) and rediscovered in the context of microbiome studies (Lovell et al., 2011; Friedman and Alm, 2012; Lovell et al., 2015; Kurtz et al., 2015; Morton et al., 2017). Unfortunately, the effect cannot be diluted away as has been recommended (Weiss et al., 2016). Understanding that there is a correlation problem is crucial, since unconstrained correlation or covariation are key concepts for ordination, clustering, network analysis and differential (relative) abundance determination. Compositional data have a negative correlation bias and a different correlation structure than the underlying count data. Even worse, compositional data exhibit spurious correlation upon subsetting or aggregation. The “Correlation” section in the Supplement shows that correlation is not a reliable or a reproducible indicator of the underlying data when dealing with compositional data.

Finally, differential (relative) abundance measures do not account for compositionality (Fernandes et al., 2013; Mandal et al., 2015; Gloor et al., 2016a). Large scale tool benchmarking has revealed that differential (relative) abundance tools in common use are sensitive to sparsity (Thorsen et al., 2016) and consequently exhibit unacceptably high false positive identification rates (Hawinkel et al., 2017).

In summary the analysis of compositional data using current protocols has several challenges. However, as shown below these issues can be addressed in a satisfactory way using tools that account for the compositional nature of the data.

## 3. Analysis of HTS using CoDa methods

Compositional datasets from HTS can be analyzed in a rigorous manner by adapting tools from other fields (Van den Boogaart and Tolosana-Delgado, 2013; Pawlowsky-Glahn et al., 2015) and using new tools based on the same underlying foundations (Fernandes et al., 2013; Erb and Notredame, 2016; Silverman et al., 2017; Quinn et al., 2017). There are now examples in the literature that provide guidance on how to do some or all of these analyses on HTS datasets, including meta-transcriptomics (Macklaim et al., 2013) and tag-sequencing (McMurrough et al., 2014; Bian et al., 2017). We briefly review the approaches below.

The starting point for any compositional analyses is a ratio transformation of the data. Ratio transformations capture the relationships between the features in the dataset and these ratios are the same whether the data are counts or proportions. Taking the logarithm of these ratios, thus log-ratios, makes the data symmetric and linearly related, and places the data in a log-ratio coordinate space (Pawlowsky-Glahn et al., 2015). Thus, we can obtain information about the log-ratio abundances of features *relative to other features* in the dataset, and this information is directly relatable to the environment. We cannot get information about the absolute abundances since this information is lost during the sequencing process as explained in Figure 1. However, log-ratios have the nice mathematical property that their sample space is real numbers, and this represents a major advantage for the application of standard statistical methods that have been developed for real random variables.

Often the centered log-ratio (clr) transformation introduced by Aitchison (1986) is used. Given an observation vector of *D* “counted” features (taxa, operational taxonomic units or OTUs, genes, etc.) in a sample, **x** = [*x*_1_, *x*_2_, …*x*_*D*_], the clr transformation for the sample can be obtained as follows:

(1) $$\begin{array}{rcl} {\text{x}}_{clr} & = & \left[log\left(x_{1}/G\left(\text{x}\right)\right),log\left(x_{2}/G\left(\text{x}\right)\right)\ldots log\left(x_{D}/G\left(\text{x}\right)\right)\right],\\ G\left(\text{x}\right) & = & \;\sqrt[{D}]{x_{1}\cdot x_{2}\cdot \ldots \cdot x_{D}} \end{array}$$

*G*(**x**) is the geometric mean of **x**. The clr transformed values can be used as inputs for multivariate hypothesis testing using tools such as MANOVA, regression etc. (Van den Boogaart and Tolosana-Delgado, 2013) and for model building. The clr-transformed values are scale-invariant; that is the same ratio is expected to be obtained in a sample with few read counts or an identical sample with many read counts, only the precision of the clr estimate is affected. This is elaborated in the “Probability” and “Log-ratio transformations” section in the Supplement, but the consequence is that count normalization is unnecessary and indeed, undesirable since information on precision is lost.

The *G*(**x**) cannot be determined for sparse data without deleting, replacing or estimating the 0 count values. Fortunately, there are acceptable methods of dealing with 0 count values as both point estimates using zCompositionsR package (Palarea-Albaladejo and Martín-Fernández, 2015), and as a probability distribution using ALDEx2 available on Bioconductor. Converting the single estimate to a probability vector prior to clr transformation produces a scale-invariant measure since this accounts for the precision of the estimate of the probabilities for each feature; we refer advanced readers to the more technical literature (Jaynes and Bretthorst, 2003; Fernandes et al., 2013; Gloor et al., 2016a) and the “Probability” section of the Supplement for more information.

There are compositional replacements for distance determination that is used for clustering and ordination. The first is the philr phylogenetic transform (and R package) based on balances (binary partitions) along an evolutionary tree (Silverman et al., 2017) that is a replacement for the familiar UniFrac distance metric. Distances determined by phylogenetic transforms have the advantage that the binary partitions chosen have a simple interpretation and the correlation structure of the data is fully accounted for. However, the disadvantage is that only the relationships between the chosen partitions can be examined. A second distance metric is the Aitchison distance, which is simply the Euclian distance between samples after clr transformation, and the distances between samples are the same as the phylogenetic ilr. The Aitchison distance is superior to both the widely used Jensen-Shannon divergence and the Bray-Curtis dissimilarity metrics, being more stable to subsetting and aggregating of the data, and being a true linear distance (Aitchison et al., 2000).

The replacement for β-diversity exploration of microbiome data is the variance-based compositional principal component (PCA) biplot (Aitchison, 1983; Aitchison and Greenacre, 2002) where the relationship between inter-OTU variance and sample distance can be observed (Gloor et al., 2016b). The compositional biplot has several advantages over the principal co-ordinate (PCoA) plots for β-diversity analysis. The results obtained are very stable when the data are subset (Bian et al., 2017), meaning that exploratory analysis is not driven simply by the presence absence relationships in the data nor by excessive sparsity (Wong et al., 2016; Morton et al., 2017). PCA plots can be substantially more reproducible, since they do not depend upon an presumed underlying tree that may need to be regenerated with each data subset, or when new taxa need to be incorporated. This simplicity facilitates exploratory data analysis. Compositional PCA biplots display the relationships between OTUs and the distances between samples on a common plot. It is possible to glean substantial qualitative information regarding the quality of the dataset and the relationships between groups with this tool (Aitchison and Greenacre, 2002; Gloor et al., 2016b), and examples are shown in the “Biplot” section of the Supplement.

As noted above, the correlation is unreliable in compositional datasets because of the negative correlation bias and the instability of correlation to subsetting the data. This is explained more fully in the supplement (Pearson, 1897; Aitchison, 1986) but these problems are observed with all non-compositional correlation methods (Ortego and Egozcue, 2013). Unfortunately, correlation cannot be subjected to a principled process to determine the optimal method as has been advocated recently (Weiss et al., 2016).

There are several more rigorous approaches that can be applied to analyze correlation in microbiome datasets, including SPARCC (Friedman and Alm, 2012) and SPieCeasi (Kurtz et al., 2015), both of which assume a sparse data matrix, and the ϕ (Lovell et al., 2015) and ρ (Erb and Notredame, 2016) metrics (the published versions of which required a non-sparse matrix). These latter two metrics have been incorporated into the R package propr, that includes an adaptation allowing the calculation of the metrics with sparse data that gives an expected value of ρ (E(ρ)), that approaches 1 if the two features have exactly constant ratios in the data (Lovell et al., 2015; Quinn et al., 2017). Supplementary Figure 2 shows that the expected value of ρ is much more stable to subsetting than are familiar correlation metrics, and becomes more reproducible as the value of E(ρ) approaches 1, thus indicating greater precision in estimation as correlation becomes stronger. However, determining an optimal and general approach for correlation in compositional datasets is an open research problem. Supplementary Figures 2–5 have a more extended explanation of the correlation problem and the use of E(ρ) as a proposed solution.

Differential (relative) abundance of OTUs between groups in compositional data is often examined using purpose-built tools that compare the difference in relative abundance across samples, and recently tools adapted from the domain of RNA-seq have been suggested. Unfortunately, these approaches do not account for the compositional nature of the data, and so can be particularly sensitive to the negative correlation bias and large variability of such datasets (Fernandes et al., 2013). Indeed benchmarking suggests that traditional tools exhibit different false positive rates with different levels of sparsity (Thorsen et al., 2016), and that the false positive rates can be up to 20× higher than expected (Hawinkel et al., 2017).

Tools based on an approximate compositional foundation are available. The ANCOM tool performs statistical tests on point estimates of data transformed by an additive log ratio, where (presumed) invariant taxa are chosen as the denominator (Mandal et al., 2015). ANCOM is being incorporated into the popular QIIME suite of microbiome analysis tools (Weiss et al., 2017). The ALDEx2 tool performs statistical tests on the clr values from a modelled probability distribution of the dataset (Supplementary data Equations 1–4), and reports the expected values of parametric and non-parametric statistical tests along with effect-size estimates. This approach reduces the false-positive identification problem to near 0 in real and modelled microbiome datasets with little effect of sensitivity (Thorsen et al., 2016) and is observed to be relatively insensitive to change when the data are subset (Fernandes et al., 2014). There are many examples in the literature on its use (Macklaim et al., 2013; McMurrough et al., 2014; Bian et al., 2017) and in the Supplementary.

In summary, the analysis of compositional data by traditional methods can appear to give satisfactory results. However, these results can be misleading and unpredictable. Compositionally-appropriate tools exist as drop-in replacements at each stage of the analysis as shown in Figure 2, and interested readers are directed to the supplementary and to other published examples (Macklaim et al., 2013; Fernandes et al., 2014; McMurrough et al., 2014; Lovell et al., 2015; Mandal et al., 2015; McMillan et al., 2015; Gloor and Reid, 2016; Gloor et al., 2016b; Bian et al., 2017; Silverman et al., 2017; Quinn et al., 2017), and the similar correspondence analysis implemented in the phyloseq package (McMurdie and Holmes, 2013).

## Author contributions

GG conceived and wrote the initial draft of the manuscript. JM conceived and made Figures 1, 2. JM, JE, and VP-G edited the draft. All authors agreed with the contents of the final version.

### Conflict of interest statement

The authors declare that the research was conducted in the absence of any commercial or financial relationships that could be construed as a potential conflict of interest.
