# Supplementary material for: Microbiome Datasets Are Compositional: And This Is Not Optional
Source: Front Microbiol. 2017 Nov 15;8:2224. doi: 10.3389/fmicb.2017.02224 (PMC5695134; doi:10.3389/fmicb.2017.02224)
Supplement: Supplementary file 1 [file Presentation1.PDF]

Supplementary workflow

Greg Gloor

27 October, 2017

Contents

About this document **1**

Reproducing the analysis 1

R packages required 1

Standard and compostional workflow **2**

Log-ratio transformations **3**

The compositional PCA biplot **4**

Correlation **6**

The problem of spurious correlation 6

Numbers and proportions are very different . . 6

Spurious correlation in action 7

Geometric intuition of correlation of compositional data 8

The negative correlation bias 8

The problem of positive correlations 8

Interpretation of Figure 7 8

Probability and expected values **9**

Imputing 0 9

Why expected values are useful 9

The expected value ρ: $E(\rho)$: 10

Differential relative abundance with ALDEx2 . 12

Summary **13**

References **14**

About this document

This document is an .Rmd document and can be found at:

github.com/ggloor/paper_supplements/Frontiers_CoDa_2017

The document is the supplement and companion to the "Microbiome datasets are compositional: and this is not optional." review article. This document is necessarily more technical than the review. It contains interspersed markdown and R code that is compiled into a pdf document and supports the figures and assertions in the main article. R code is not exposed in the pdf document but is referred to by `R code block` in the text so that the interested reader can work through the example code themselves.

Reproducing the analysis

From an R command prompt you can compile this document into PDF if you have LaTeXand pandoc installed:

`rmarkdown::render('Frontiers_supplement.Rmd')` or you can open the file in RStudio and compile in that environment.

We will use a subset of the Human Microbiome Project (HMP) oral microbiome dataset and will be comparing samples from attached keratinized gingiva (ak) with outer plaque biofilm (op) included in the CoDaSeq R package. The HMP dataset is exceedingly sparse (contains many 0 values), and has wide variation in read depth: this makes the dataset a good stress test for the method.

R packages required

We will need the following R packages and add-ons (`R_block_1`).

1. knitr (CRAN)
2. CoDaSeq (https:github.com/ggloor/CoDaSeq)
3. ALDEx2 (Bioconductor)
4. zCompositions (CRAN)
5. igraph (CRAN)
6. grDevices (CRAN)
7. car (CRAN, loaded with ALDEx2)
8. propr (CRAN)
9. vegan (CRAN)

Standard and compostional workflow

Operation	Standard approach	Compositional approach
Normalization	Rarefaction<br>'DESeq'	CLR<br>ILR<br>ALR
Distance	Bray-Curtis<br>UniFrac<br>Jenson-Shannon	Aitchison
Ordination	PCoA<br>(Abundance)	PCA<br>(Variance)
Multivariate comparison	perManova<br>ANOSIM	perMANOVA<br>ANOSIM
Correlation	Pearson<br>Spearman	SparCC<br>SpiecEasi<br>ϕ<br>ρ
Differential abundance	metagenomSeq<br>LEfSe<br>DESeq	ALDEx2<br>ANCOM

Figure 1: Standard and compositional methods to analyze the microbiome.

The standard microbiome analysis, illustrated in Figure 1, is initiated by normalizing the reads to a common sequencing depth. Historically normalization was performed by rarefaction, but increasingly uses the method of count normalization from the DESeq package (Weiss et al., 2017). The results of the standard analysis pipelines strongly depend upon the read depth without normalization. The compositional replacement is to covert the data to ratios, generally using the centred log-ratio but other approaches are possible (Aitchison, 1986; Gloor et al., 2016c). Downstream methods using a log-ratio approach are affected minimally by read depth, unless the difference in depth between samples is egregious. The usual next step is to determine distances between samples for β-diversity, ordination and multivariate comparison. The standard approach offers a rich choice of distance and dissimilarity metrics, largely because no single metric captures the data completely. There is considerable uncertainty regarding the appropriate metric (Weiss et al., 2017). In contrast, the compositional approach offers the Aitchison distance metric, which is an appropriate and consistent metric (Aitchison et al., 2000, Martín-Fernández et al. (1998)). This simplifies the downstream analyses and prevents 'distance-metric hacking' whereby different metrics are used for different purposes (Wong et al., 2016). Exploratory data analysis and multivariate comparison between groups in the standard approach uses a Principle Co-ordinates Analysis which is based on identifying relationships between samples using the distance metric(s) calculated in the previous step. PCoA can be very powerful, but care must be taken to ensure that the results are not simply driven by the most abundant taxon, gene or OTU in the analysis (Gorvitovskaia et al., 2016). In contrast, ordination in the compositional approach is driven by the genes, OTUs or taxa that have the largest variation in the dataset (Aitchison and Greenacre, 2002). Furthermore, plotting using PCA biplots has the added benefit of displaying the relationships between the samples and the features in the samples on one plot. In a compositional analysis the Aitchison distance can be used as a drop-in replacement in the multivariate comparison step. Any of the standard correlation strategies are wrong for these data (Lovell et al., 2015), and there are a number of methods described below that may be useful. We suggest the use of the ρ metric (Erb and Notredame, 2016), or other compositional approaches. Many tools are available to estimate which features exhibit differential relative abundance in these datasets. They have many different characteristics and often generate many false positives, but the compositionally appropriate ANCOM and ALDEx2 are generally less prone to these problems than are the non-compositional approaches (Thorsen et al., 2016; Weiss et al., 2017)

Log-ratio transformations

There are three main log-ratio transformations; the additive log-ratio (alr), centred log-ratio (clr) and the isometric log-ratio (ilr) (Pawlowsky-Glahn et al., 2015).

Given an observation sample vector $\vec{\boldsymbol{x}}$ of D 'counted' features (taxa, operational taxonomic units or OTUs, genes, etc.) $\vec{\boldsymbol{x}} = [x_1, x_2, ...x_D]$:

The alr is the simply the elements of the sample vector divided by a presumed invariant feature, which by convention here is the last one:

$$\vec{\boldsymbol{x}}_{alr} = [log(x_1/x_D), log(x_2/x_D) \ldots log(x_D - 1/x_D] \quad (1)$$

This is similar to the concept used in quantitative PCR, where the relative abundance of the feature of interest is divided by the relative abundance of a (presumed) constant 'houseskeeping' feature. Of course there are two major drawbacks. First, that the experimentalist's knowledge of which, if any, features are invariant is necessarily incomplete. Second, is that the choice of the (presumed) invariant feature has a large effect on the result if the presumed invariant feature is not invariant, or if it is correlated with any other features in the dataset. Interestingly, an early proposal was to use the geometric mean of a number of internal controls (Vandesompele et al., 2002), leading to the next transformation.

The centered log-ratio (clr) transformation introduced by (Aitchison, 1983,Aitchison (1986)) uses the geometric mean of all features as the denominator:

$$\vec{\boldsymbol{x}}_{clr} = [log(x_1/G(\vec{\boldsymbol{x}})), log(x_2/G(\vec{\boldsymbol{x}})) \ldots log(x_D/G(\vec{\boldsymbol{x}}))], \\ G(\vec{\boldsymbol{x}}) = \sqrt[D]{x_1 \cdot x_2 \cdot ... \cdot x_D} \quad (2)$$

where $G(\vec{\boldsymbol{x}})$ is the geometric mean of $\vec{\boldsymbol{x}}$.

The clr is often criticized since it has the property that the sum of the clr vector must equal 0. This constraint causes a singular covariance matrix; i.e., the sum of the covariance matrix is always a constant (Pawlowsky-Glahn et al., 2015). However the clr has the advantage of being readily interpretable, a value in the vector is its abundance *relative* to a mean value.

The ilr is the final transformation, and is a series of sequential log-ratios between two groups of features. For example, the philr transformation is the series of ratios between OTUs partitioned along the phylogenetic tree (Silverman et al., 2017), although any other sequential binary partitioning scheme is also possible (Pawlowsky-Glahn et al., 2015). The ilr transformation does not suffer the drawbacks of either the alr or clr, but does not allow for insights into relationships between single features in the dataset. Nevertheless, ilr transformations permit the full-range of multivariate tools to be used, and are recommended whenever possible.

The ilr and clr are directly comparable in a two important ways: First, the distances between samples computed using an ilr and clr transformation are equivalent. Second, the clr approaches the ilr in other respects as the number of features becomes large. In this respect, the large number of features — hundreds in the case of OTUs, thousands in the case of genes — in a typical experiment works in our favour. Thus, while not perfect, the clr is the most widely used transformation. However, care must be taken when interpreting its outputs since single features must always be interpreted as a ratio between the feature and the denominator used for the clr transformation. The problems of using clr are apparent when some subcomposition or group of taxa is analysed for further insight since the geometric mean of the subcomposition is not necessarily equal to that of the original composition, leading to potential inconsistencies.

Log-ratio values of any type do not need to be normalized since the total sum is a term in both the numerator and the denominator. Thus, the same log-ratio value will be obtained for the vector of raw read counts, or the vector of normalized read counts, or the vector of proportions calculated from the counts. Thus, log-ratios are said to be equivalence classes such that there is no information in the total count (aside from precision) (Barceló-Vidal et al., 2001). Attempts to 'open' the data are doomed to failure because the data cannot be moved from the simplex to Euclidian space. The total count delivered by the sequencing instrument is a function of the instrument and not the number of molecules sampled from the environment, thus the total count has no geometric meaning. If the data are collected in such a way that the total count represents the actual count in the environment, then the data are not compositional and issues regarding compositional data disappear. However, at present all sequencing platforms deliver a fixed-sum, random sample of the proportion of molecules in the environment.

Note that this does not mean that the read depth is irrelevant since more reads for a sample translate into greater precision when estimating the proportions (Fernandes et al., 2013,Gloor et al. (2016b)).

The compositional PCA biplot

The compositional PCA biplot (Aitchison, 1983, Aitchison and Greenacre (2002)) made by a Singular Value Decomposition of the CLR-transformed data is normally the first exploratory tool used to examine the dataset (`R_Block_2`). The quantitative information from the association and differential abundance tests can be obtained, in a qualitative manner, from the initial PCA plots themselves.

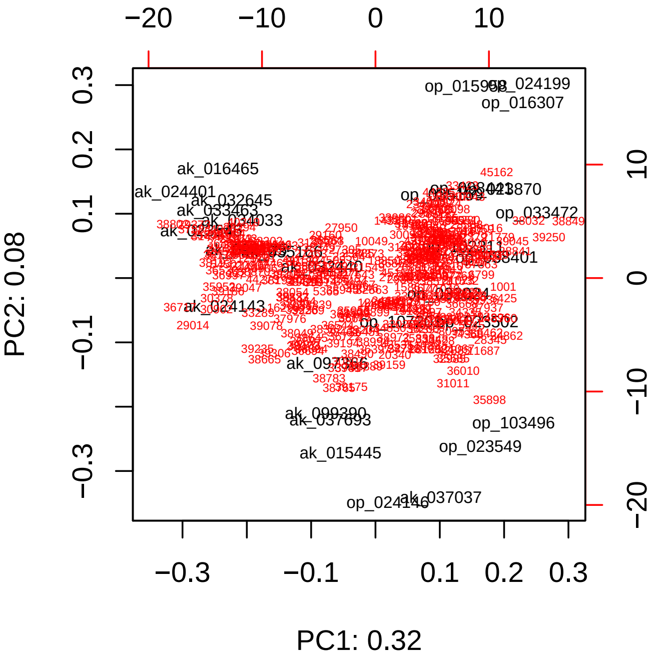

Figure 2: Compositional biplot generated from the CLR transformed OTU values with 0 replacement. The biplot is shown with scale=1, projecting the distances between samples onto the 2-D image as best as possible. The distance between OTUs (red) is a measure of their compositional association, but this is only a crude measure relative to ρ.

We have previously given extensive guidance on the interpretation of these plots for microbiome data (Gloor et al., 2016c), but in general the large numbers of variables and the small number of samples often obscures important relationships. Thus, we prefer to plot the loadings and the sample relationships separately, and functions are provided for this in the `CoDaSeq` package.

When we plot the individual OTUs separately as in Figure 3 (`R_Block_3`) on a loadings plot the resulting plot contains qualitative information on both differential relative abundance of OTUs and on association between OTUs. The distance and direction of OTU from the center of the plot is proportional to the standard deviation of the CLR value of that OTU in the dataset (Aitchison and Greenacre, 2002). In theory, OTUs that have a short link between them, (i.e., are nearly the same direction and distance from the origin) should be compositionally associated. However, our ability to make this conclusion is tempered by the proportion of variance explained by the first two principle components, and in practice at least 90% of the variance needs to be explained for a high confidence determination. Thus, the compositional PCA biplot, or the loadings plot itself, should be used mainly for display, and the numerical differential relative abundance and compositional association measures should be used for quantitative insights.

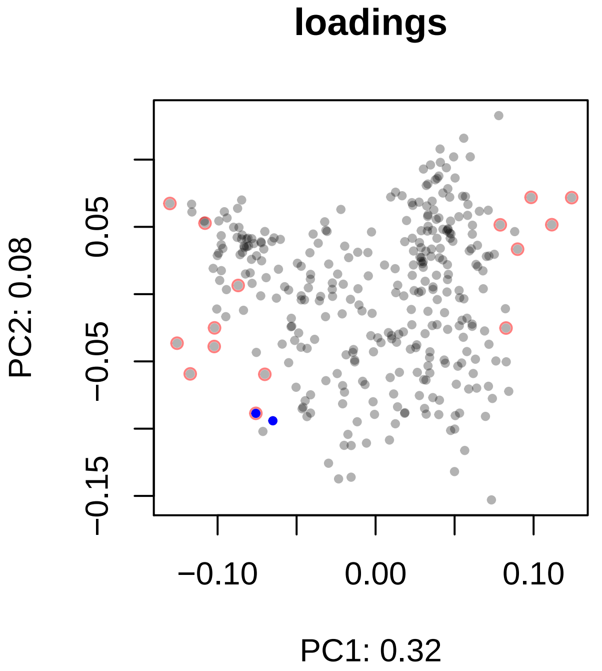

Figure 3: Plot of the relationship between the OTUs from the SVD. Here each OTU is colored red if it was identified as having an effect size greater than 1. The two OTUs with the ρ value closest to one are highlighed in blue. See later sections for an explanation of effect size and ρ.

Similarly, we can plot the individual samples in Figure 4 (`R_Block_4`), with the ak samples in red, and the op samples in blue. Here the distances between points is proportional to the Euclidian distance of the CLR vectors of the samples. This is referred to as the Aitchison distance.

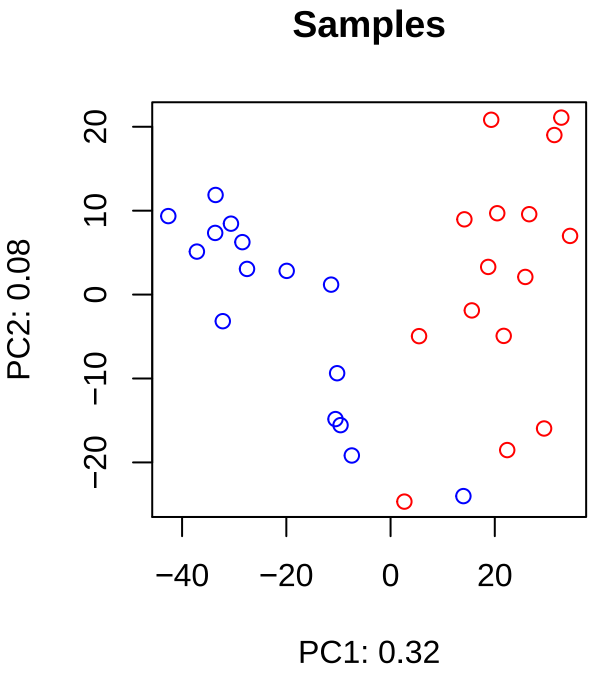

Figure 4: Plot of the individual samples from the SVD output, with the ak samples in red, and the op samples in blue. Here the distance between points is proportional to the Euclidian distance of the CLR vectors of the samples. This is referred to as the Aitchison distance.

Similarly, we can calculate an Aitchison distance matrix as in `R_Block_5` to generate the dendrogram in Figure 5 with sample names colored red or blue depending on if they fall within the op group, or the ak group. Note that op_024146 falls completely within the ak group in the dendrogram. This is in accordance with the biplot which shows the same partitioning.

The multivariate distance between samples can be estimated using the Aitchison distance and the significance calculated using the vegan `anosim` function (`R_Block_5`). That is, the Aitchison distance can be used as a drop-in replacement for other distance or dissimilarity metrics used in the microbome literature. The Aitchison distance is compatible with compositional assumptions. By this test, the op and ak samples have a significantly different composition ($P < 0.001$).

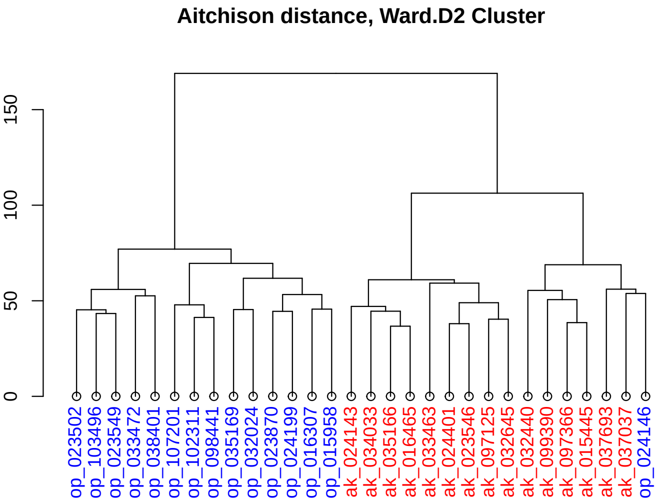

Figure 5: Dendrogram plot of the individual samples from distance matrix output. Samples from op are colored in blue, and ak are coloured in red.

Table 1: Counts

	A	B	C	D	E
s1	10	20	20	50	50
s2	15	40	30	20	200
s3	20	80	10	30	15

Table 2: Proportions

	A	B	C	D	E
s1	0.067	0.133	0.133	0.333	0.333
s2	0.049	0.131	0.098	0.066	0.656
s3	0.129	0.516	0.065	0.194	0.097

Correlation

The problem of spurious correlation

Spurious correlation arises when data are constrained by a constant denominator; that is data that are represented as proportions, percentages, probabilities, relative abundances, etc (Aitchison, 1986). Understanding the correlation problem is crucial since spurious correlation is the basis for essentially all data analysis anomalies associated with compositional data. The problem has been recognized since the beginning of statistical practice as noted by the quote from a paper by Karl Pearson [1897]:

"if the ratio of two absolute measurements on the same or different organs be taken it is convenient to term this ratio an index.

If $u = f_1(x, y)$ and $v = f_2(z, y)$ be two functions of the three variables x, y, z, and these variables be selected at random so there exists no correlation between x, z, y, z, or z, x, there will still be found to exist correlation between u and v. Thus a real danger arises when a statistical biologist attributes the correlation between two functions like u and v to organic relationship . . . ." (Pearson, 1897)

This problem exists whenever there is a constant sum or denominator in a dataset: proportion, percentage, ppm, etc, or equivalently, when the dataset cannot have an infinite sum as happens in hight throughput sequencing datasets.

Numbers and proportions are very different

Before we examine correlation in the large HMP dataset, we will illustrate the problem of correlation and compositionality in a small, easily understood dataset generated in `R_block_6` and displayed in the tables at the top of the page. Assume first that we are dealing with numbers and there are three samples (s1, s2, s3) each with five features (A-E).

Tables of numbers (Table 1), proportions calculated from the complete dataset (Table 2), and proportions calculated from the dataset with variable E removed (Table 3). This last situation is called a sub-composition and is a common operation when dealing with high throughput data. For example, rRNA and tRNA are usually removed physically or computationally prior to analysis of transcriptomic datasets, and 16S rRNA gene sequencing datasets include only those taxa that can have their DNA extracted and amplified.

Table 3: Subset Proportions

	A	B	C	D
s1	0.100	0.200	0.200	0.500
s2	0.143	0.381	0.286	0.190
s3	0.143	0.571	0.071	0.214

Note that the values represented as numbers, as proportions of the whole, and as proportions of the first 4 variables are different. For example, numerically $s2_D = 20$ and $s3_D = 30$, but proportionally these values are 0.066 and 0.194 in the whole proportional dataset and 0.19 and 0.214 in the subset proportional dataset. Thus, the *absolute difference* between values is not stable. However, the *ratios of the parts* remains intact. That is:

$$s1_A/s1_B = \frac{1}{2} = \frac{0.067}{0.133} = \frac{0.1}{0.2} = 0.5$$

as counts, proportions of the whole, and proportions of the subset..

If we calculate the correlations for feature A from the whole, from a subset, from the whole as proportions, and a subset converted to proportions we see how the correlations can be affected by these changing values in Table 4. Here we observe that the correlations between features can change in both magnitude and in sign by an alarming amount.

Table 4: Correlation metrics designed for real numerical data such as Pearson or Spearman correlation coefficients are not reliable in compositional data

	B	C	D	E
S all	1	-0.5	-0.5	-0.5
S sub	1	-0.5	-0.5	
P all	1	-0.5	0.5	-1
P sub	0.9	0	-0.9	

Spurious correlation in action

In Figure 6A, the same count data set (absolute abundances) is presented (5 features, 3 samples), and feature D is plotted vs feature A as numbers, as proportions with all features present (Fig 6B) and as proportions with feature E removed (Fig 6C). We can see how converting to proportions changes the relationships between features. This trivial example shows that absolute abundance data and the relative abundance data do not provide equal correlations on the parts in common, and thus that approaches advocated in the literature that do not account completely for compositionality can be inaccurate (McMurdie and Holmes, 2014, Weiss et al. (2017)). Later we show that the Spearman's correlation is not stable when real data are subset and that Pearson correlation is susceptible to false positives.

Figure 6 plots A vs D from the three tables and display the Spearman's and Pearson's correlation coefficients; the code is in `R_block_7`.

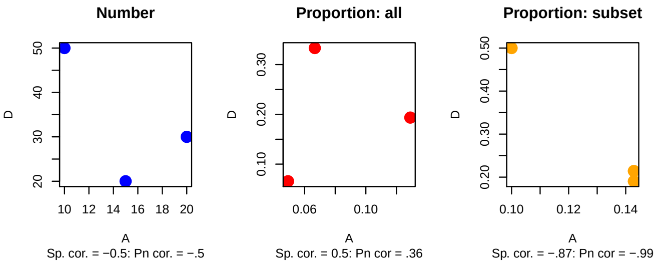

Figure 6: Plots of A vs D for numbers, proportions and proportions from the subset where the last column was dropped. We can see that the relationship between the data points is not the same for the numerical and proportional data and that the relationship changes again when the proportional data are subset. *This is spurious correlation* because we see there is an unpredictable correlation observed between two variables whenever they share a common denominator.

Geometric intuition of correlation of compositional data

We usually think of correlations as linear relationships of the type $y = mx + b$, and measure correlation coefficients as a standardized covariance relationship. However, this approach does not work when analyzing correlations of compositional data regardless of the transformation (Lovell et al., 2015).

The negative correlation bias

The variables in compositional data have a negative correlation bias. This is obvious in the case of a coin toss, where intuitively we know that the the observation of a fraction of heads, h must be associated with a fraction of tails equal to $1 - h$. This also occurs in the multivariate case, where any observation of the fraction of the number 6, s, from a number of rolls of a die, must correspond with the fraction of the other rolls of $1-s$. Thus there must be at least one and possibly many negative correlations **because of the structure of compositional data**, the problem is that there is no theoretical method to distinguish those negative correlations that arise structurally from those that are true negative correlations driven by the underlying process (Lovell et al., 2015).

The problem of positive correlations

Positive correlations are generally observed whenever two features have a simple linear relationship. However, compositional data must have a constant ratio relationship to be correlated (Lovell et al., 2015), and this is a subset of what would be discovered using Pearson or Spearman correlations.

Thus, when examining compositional data two variables (or groups of variables) must have a constant ratio relationship in Euclidan space. In other words, if we plot x and y on a scatter plot they will lay on a line projecting from the origin. Such a line fulfils the linear model $y = mx$. We can see this in the Figure 7 "Euclidian" where the red and the black variables have a line of best fit that pass through the origin. However the blue variables have an intercept of 20. All three of these have an equivalently high Pearson and Spearman correlation coefficient. In the "Log" plot, the red and black points fall on lines with slope 1, but the blue line does not. Thus, while the blue variables have a high correlation, they are not compositionally associated since the ratio is not constant (Lovell et al., 2015). The code for Figure 7 is in `R_block_8`.

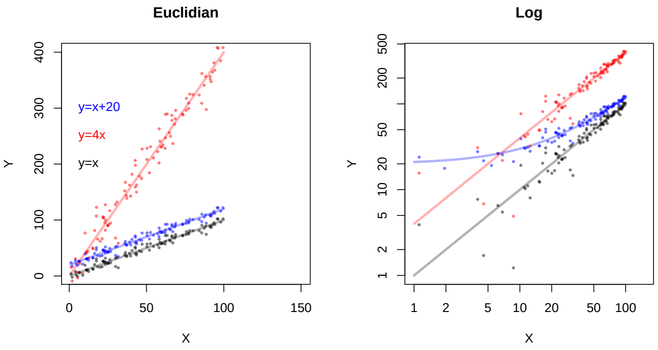

Figure 7: The Euclidian and Log plots show that data on a line with an intercept do not maintain a constant ratio. Two variables (in red or black) that have a constant ratio will be linear and pass through the intercept in Euclidian space, and will have a slope of 1 on a log-log plot, we can see that the blue line with an intercept of 2 is neither linear, nor does it have a slope of 1.

Interpretation of Figure 7

1. note that any pairs of variables that have a constant ratio appear on a line projecting from the origin in Euclidian space. The red and black variables are in constant ratio, but the blue variables are not.
2. Constant ratio relationships cannot have an intercept in Euclidan space plots because the constant ratio between features is not preserved. This becomes obvious as a non-linear relationship in log-space
3. Constant ratio relationships can have a slope $\neq 1$ in Euclidian space, the slope becomes the intercept when plotted on a log-log scale.
4. Familiar measures of correlation do not require an intercept of 0
5. False positive correlations are thus an issue when observing either positive and negative correlation but for different reasons.

Probability and expected values

Imputing 0

High throughput sequencing data appears to deliver counts per genetic fragment in each sample (the genetic fragment can be a targeted amplicon or a short RNA or DNA sequence fragment). **However, this is a myth: the sequencing instrument can only deliver a fixed number of sequence reads**, and so the data are constrained to a constant, arbitrary sum.

The process that generates the reads is to take a random sample from the environment, make a library and take a random sample of the library and sequence it. The number of molecules in the environment and in the library is substantially greater than the number of molecules sequenced, making sequencing a multivariate Poisson sampling process (Fernandes et al., 2013). Thus, it is more proper to think about each count as a probability p of observing the count for each genetic fragment conditioned on the total sequencing depth and the underlying frequency of the molecules in the environment. That is: for the jth random vector of counts from the environment, $\vec{s}_j$:

$$\vec{s}_j = [s_1, s_2 \ldots s_D]$$

we wish to determine the underlying frequency f_{ij} of the ith molecule in the jth environment, which is proportional to the probability of sampling the ith molecule from that environment. A vector of the maximum likelihood estimates of the underlying probabilities is:

$$\vec{p}_j = [p_1 \ldots p_D] = \frac{\vec{s}_j}{\alpha_j}; \alpha_j = \sum \vec{s}_j \tag{3}$$

$$\vec{p}_{ij} = \frac{(\vec{s}_{ij}|f_{ij})}{\alpha_j} \tag{4}$$

The uncertainty of measurement of $\vec{p}_{ij}$ depends directly on f_{ij} and inversely with α_j. As f_{ij} approaches or becomes smaller than $1/\alpha_j$ (i.e., as $\vec{s}_{ij} \to 0$), the uncertainty of $\vec{p}_{ij}$ becomes very large indeed (Jaynes and Bretthorst, 2003, Fernandes et al. (2013)). Thus, the maximum likelihood estimate of the probability vector (and the corresponding count vector) can be exponentially wrong when the data are sparse (Newey and McFadden, 1994). Indeed, we observe a very large amount of variation in technical replicates (Fernandes et al., 2013, Gloor et al. (2016b)).

Instead of attempting to identify a point estimate of the probability vector, we adapt standard Bayesian approaches to estimate the probabilities by making the assumption that the nucleotide fragments are derived as a multivariate Poisson random sample of the underlying environment (Fernandes et al., 2013). Within this framework we generate k random instances of the probabilities by drawing from the Dirichlet distribution to generate an estimate of the posterior distribution of the underlying probabilities.

$$P_{j,1\ldots k} = \begin{pmatrix} \vec{p}_{j1} \\ \vec{p}_{j2} \\ \vdots \\ \vec{p}_{jk} \end{pmatrix} = \begin{pmatrix} p_{11} & p_{21} & p_{i1} & \cdots & p_{D1} \\ p_{12} & p_{22} & p_{i2} & \cdots & p_{D2} \\ \vdots & \vdots & \vdots & \ddots & \vdots \\ p_{1k} & p_{2k} & p_{ik} & \cdots & p_{Dk} \end{pmatrix} \sim Dirichlet_{(1\ldots k)}(\vec{s}_j + 0.5) \tag{5}$$

Each of the k random instances of the posterior distribution is a probabilistic estimate of the data, given the observed vector of counts $\vec{s}_j$. In the case where the $\vec{s}_{ij} = 0$, $P_{i,j,1\ldots k}$ contains many non-0 estimates of the probability of $\vec{s}_{ij}$ *each of which is an equally valid estimate of the probability of that value.* Similarly, if $\vec{s}_{ij}$ is non-0, $P_{i,j,1\ldots k}$ contains many estimates of that probability.

A distribution of centered log-ratio values $C_{j,1\ldots k}$ can now be calculated since all values are non-0, these are calculated row-wise, i.e. for the kth row:

$$C_{jk} = log(p_{i,j,k}) - mean(log(P_{jk})) \tag{6}$$

At this point, each value in the vector $\vec{s}_j$ has been used to generate a distribution of centered log-ratio (CLR) values where no log-ratio calculation included a 0 value, but a value of 0 could approach 0 with an arbitrary probability. Thus, the distribution of CLR values are wide near the low count margin, and when α is small, and become progressively narrower otherwise. While the Dirichlet distribution is not a good null model for compositional data because it makes strong assumptions of independence (Aitchison, 1986), it is an adequate model to generate the posterior distribution of probabilities derived from count compositional data (Fernandes et al., 2013, Gloor et al. (2016b)).

Why expected values are useful

Any univariate test statistic of OTUs or genes (differential relative abundance, compositional association, etc) or multivariate statistic can be calculated across the distribution and an expected value derived. Since the distribution is wide at the low count margin, there is a large stochastic effect on the calculated test statistic, and the expected value will tend towards the middle of the distribution of that test statistic (i.e, a p-value will tend towards a value of 0.5). Conversely, since the distribution is narrow when $\vec{s}_{ij}$ is not trivial, the expected value of the test statistic will have a smaller stochastic influence.

The expected value ρ: $E(\rho)$:

The ρ metric is a replacement for a correlation coefficient, and is based on scaling the variance matrix, an approach first conceived by (Lovell et al., 2015). Lovell et. al, suggested ϕ, where 0 denotes perfect association.

$$\phi_{xy} = \frac{Var(clr(x) - clr(y))}{Var(clr(x))}, \in 0, +\infty \tag{7}$$

or the geometric equivalent:

$$\phi_{xy} = 1 + m^2 - 2m|r| \tag{8}$$

where m is the slope and r is the correlation coefficient.

ϕ_{xy} can be modified to be symmetrical and scaled by a modification of the formula and the new metric is called ρ (Erb and Notredame, 2016):

$$\rho_{xy} = 1 - \frac{Var(clr(x) - clr(y))}{Var(clr(x)) + Var(clr(y))}, \in -1, 1 \tag{9}$$

or the geometric equivalent:

$$\rho_{xy} = \frac{2r}{m + 1/m} \tag{10}$$

The ϕ and ρ metrics are expected to be subcompositionally coherent, that is, either metric is expected to have the same value for pairs of features in common if the entire dataset is examined or any reasonable subset of the OTUs in the dataset is examined. In the context of high throughput sequencing, this expectation is only true for non-sparse data. However, the expectation may not be true for sparse data where 0 values must be imputed, or if extremely small numbers of OTUs are examined. Figure 8 shows four measures of association between two OTUs with all others that are present in both an entire and a subset dataset drawn from the HMP oral dataset. Note that the Spearman correlation coefficient is not consistent and that the point estimate of ρ is much more consistent than is the Spearman correlation coefficient. The point estimate of ρ is not identical because of sparsity; the point estimate of 0 is not identical between the whole dataset and the subset. However, observe that the $E(\rho)$ is essentially consistent in the whole dataset when compared to the subset dataset. This reproducibility becomes more consisent with more Dirichlet Monte Carlo Replicates, and the reader can modify the code in `R_block_1 and R_block_9` to demonstrate that increasing the number of DMC intances 100-fold will result in a much tighter E(ρ) relationship, although at a cost of time to calculate. The `propr` documentation contains additional information on how to interpret the results. `R_block_1` contains the calculations and `R_block_9` contains the plotting commands for Figure 8.

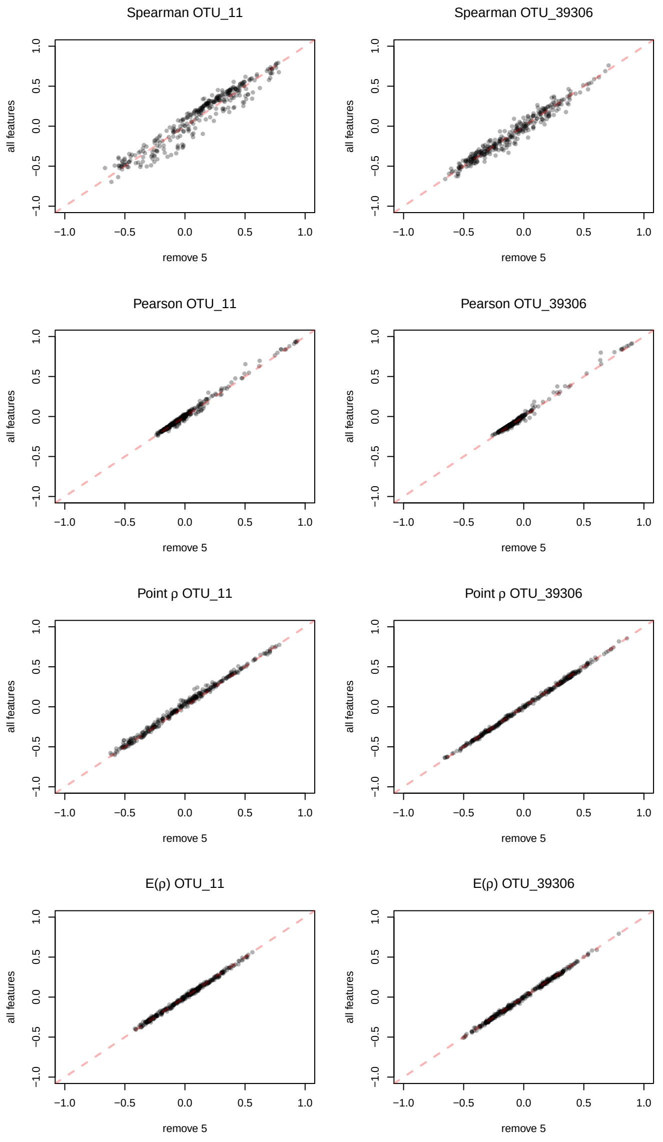

Figure 8: Measures of association should be stable to subsetting the data. The Spearman and Pearson correlation coefficients, point estimates of ρ and E(ρ) are shown for OTU number 11 or OTU number 39306 to all other OTUs calculated on log-ratio transformed values (clr), from the subset of the HMP oral microbiome dataset. The all features axis contains all OTUs in the calculation and the remove 5 axis is the same calculation performed with five OTUs removed. E(ρ) was calculated with 256 Dirichlet Monte-Carlo instances. We see that the Spearman correlation coefficient is unstable even in a dataset containing hundreds of OTUs, just like it was in Figure 6. The Pearson correlation coefficient is also unstable, but less so, and is strongly biased towards having a few extremely high correlation values, most of which will be false positives for the reasons outlined in Figure 7 and illustrated in Figure 9.

Table 5: The E(ρ) metric for selected OTU pairs.

OTU 1	OTU 2	E(rho)_all	E(rho)_r5
38349	26584	0.684	0.691
30378	29014	0.669	0.678
38802	31478	0.689	0.674
38193	35952	0.69	0.689
39306	39235	0.792	0.791

At present we must examine the linear relationship between two variables when a value of ρ is not trivially near to 1. Table 5 shows five association pairs with high E(ρ) values. Note that the expected value is similar in the complete and remove 5 dataset. The ρ metric is attempting to summarize both the slope and linearity of any relationship between the values into one number, so that a better Pearson correlation coefficient can be offset by a poorer fit to a linear line of slope 1. Figure 9 plots several associations, from Table 5 to show the slope and scatter around the line of best fit. We see that the associations between the OTUs given as examples in Table 5 are good fits to the assumption. `R_block_10` contains the code to generate the table, `R_Block_11` contains the code to generate Figure 9.

Figure 8 demonstrated that the Pearson correlation also appeared to be reproducible in compositional data. However, as noted above, the Pearson correlation can include many false positive correlations. The bottom two panels in Figure 9 plot the relationship between the Pearson r value and the E(ρ) values for the HMP dataset, and the slope and association for one pair. Here we observe that there are many high r values with modest or low E(ρ) values, and the particular one shown in red in the bottom left panel is plotted in the bottom right panel. Thus, we find that a high Pearson r value can be a false positive since the two OTUs are not necessarily in constant ratio.

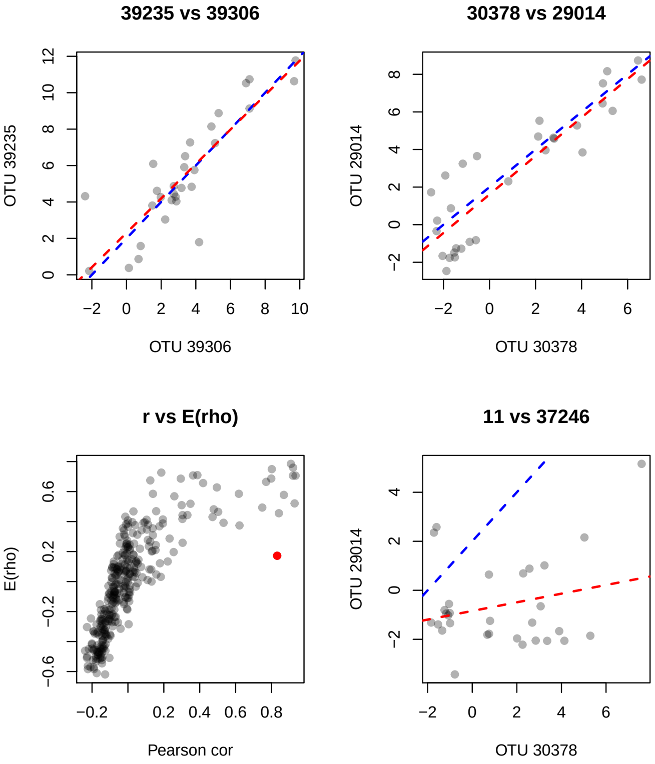

Figure 9: Plots showing the associations between two OTU pairs with the ρ value taken from the table (top two panels), and one OTU pair that has a high Pearson correlation coefficient but low E(ρ) value (bottom two panels). The dashed blue lines show the ideal line of slope 1, the dashed red lines show the line of best fit to the data. The '11 vs. 37246' pair is correlated but is not compositionally associated and the relationship between the Pearson correlation and E(ρ) and Pearson correlation for this pair of variables is highlighted in the bottom left panel. The association between these OTUs plotted in the bottom right panel, showing that this association is a false positive identified by Pearson correlation.

Differential relative abundance with ALDEx2

ALDEx2 measures differential (relative) abundance as calculated by an expected p-value, an expected Benjaminin-Hochberg adjusted p-value and as an expected standardized effect size (Fernandes et al., 2014, Gloor et al. (2016a)). The latter is a much more robust estimate, and should be used whenever possible since a standardized effect size is a much more reproducible metric of 'significance' than is a p-value (Halsey et al., 2015).

We use effect plots for display purposes because they show the relationship between difference and dispersion (Gloor et al., 2016a) which are the constituents of p-values, but are not scaled by the number of samples. We can see in Figure 10 that the majority of OTUs in the HMP dataset have much more dispersion (within group variation, analogous to the standard deviation) than they do between group difference. In fact, the majority of OTUs have a dispersion value greater than $2^3 = 8$ but less than 4-fold different relative abundance between groups. Clearly, even a low p-value is meaningless in this situation. The OTUs with greater difference than dispersion are indicated by the red points in the plot. The second plot, E vs. P, shows the relationship between the standardized effect and p-value. `R_Block_12` contains the code for Figure 10, values were calculated in `R_block_1`.

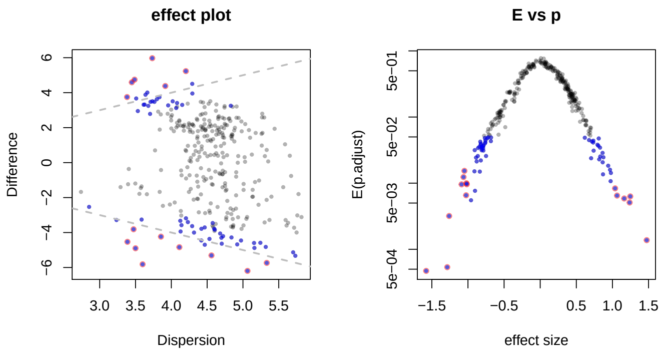

Figure 10: The effect plot shows a scatter plot of the difference between the relative abundance of OTUs in groups 1 and 2 plotted vs. the maximum dispersion of the OTU in either group: each point is an individual OTU. We can see that the dispersion for most OTUs is much greater than the difference between groups, essentially indicating that the variation within each group is larger than the difference between groups. Points are colored in blue if the expected Benjamini-Hochberg false discovery rate (FDR) value is less than 0.05, and circled in red if the absolute expected standardized effect size is ≥ 1. The E vs p plot shows the relationship between effect size and the false discovery rate. The effect size will be relatively stable regardless of the sample size, while the FDR will depend upon sample size.

Table 6: OTU genera with large effect size differences.

Veillonella
Actinomyces
unk
unk
Neisseria
Neisseria
Gemella
Lautropia
Veillonella
Selenomonas
Actinobacillus
Capnocytophaga
Streptococcus
Prevotella
Prevotella

The table shows differentially abundant OTUs in this dataset with an absolute effect size ≥ 1 and the genera they belong (`R_Block_13`):

The ALDEx2 documentation has additional information on the meanings of the test statistics and how to access and explore the data.

Summary

- compositional data are any positive data in which ratios between components are relevant. The sum of components can be constant along the sample or it can be irrelevant.
- it is useful to think of the count associated with an OTU or gene obtained through high throughput sequencing data as a probability conditioned on the underlying frequency in the environment scaled by the total read depth of the sample
- data generated by high throughput sequencing are compositional because the machine constrains the total count
- the relationship between the parts (OTUs, genes) is the only information available
- the data cannot be opened and so the data lay on a simplex: i.e., the data cannot be returned to the same space as the observations in the underlying environment
- a Bayesian estimate of the frequency can be used to generate expected values of test statistics
- any simplex is always equivalent to the unit simplex
- "in the absence of any other information or assumptions, correlation of relative abundances is just wrong" (Lovell et al., 2015). Thus we must examine two numbers, slope and correlation (Egozcue, submitted)

References

Aitchison, J. (1983). Principal component analysis of compositional data. *Biometrika* 70, 57–65.

Aitchison, J. (1986). *The statistical analysis of compositional data*. London, England: Chapman & Hall.

Aitchison, J., and Greenacre, M. (2002). Biplots of compositional data. *Journal of the Royal Statistical Society: Series C (Applied Statistics)* 51, 375–392.

Aitchison, J., Barceló-Vidal, C., Martín-Fernández, J. A., and Pawlowsky-Glahn, V. (2000). Logratio analysis and compositional distance. *Mathematical Geology* 32, 271–275. doi:10.1023/A:1007529726302.

Barceló-Vidal, C., Martín-Fernández, J. A., and Pawlowsky-Glahn, V. (2001). Mathematical foundations of compositional data analysis. in *Proceedings of IAMG*, 1–20.

Erb, I., and Notredame, C. (2016). How should we measure proportionality on relative gene expression data? *Theory in Biosciences* 135, 21–36.

Fernandes, A. D., Macklaim, J. M., Linn, T., Reid, G., and Gloor, G. B. (2013). ANOVA-like differential expression (ALDEx) analysis for mixed population RNA-seq. *PLoS ONE* 8, e67019.

Fernandes, A. D., Reid, J. N., Macklaim, J. M., McMurrough, T. A., Edgell, D. R., and Gloor, G. B. (2014). Unifying the analysis of high-throughput sequencing datasets: Characterizing RNA-seq, 16S rRNA gene sequencing and selective growth experiments by compositional data analysis. *Microbiome* 2, 15.1–15.13. doi:10.1186/2049-2618-2-15.

Gloor, G. B., Macklaim, J. M., and Fernandes, A. D. (2016a). Displaying variation in large datasets: Plotting a visual summary of effect sizes. *Journal of Computational and Graphical Statistics* 25, 971–979. doi:10.1080/10618600.2015.1131161.

Gloor, G. B., Macklaim, J. M., Vu, M., and Fernandes, A. D. (2016b). Compositional uncertainty should not be ignored in high-throughput sequencing data analysis. *Austrian Journal of Statistics* 45, 73–87. doi:doi:10.17713/ajs.v45i4.122.

Gloor, G. B., Wu, J. R., Pawlowsky-Glahn, V., and Egozcue, J. J. (2016c). It's all relative: Analyzing microbiome data as compositions. *Ann Epidemiol* 26, 322–9. doi:10.1016/j.annepidem.2016.03.003.

Gorvitovskaia, A., Holmes, S. P., and Huse, S. M. (2016). Interpreting prevotella and bacteroides as biomarkers of diet and lifestyle. *Microbiome* 4, 15. doi:10.1186/s40168-016-0160-7.

Halsey, L. G., Curran-Everett, D., Vowler, S. L., and Drummond, G. B. (2015). The fickle p value generates irreproducible results. *Nat Methods* 12, 179–85. doi:10.1038/nmeth.3288.

Jaynes, E. T., and Bretthorst, G. L. (2003). *Probability theory: The logic of science*. Cambridge, UK: Cambridge University Press Available at: http://www.loc.gov/catdir/samples/cam033/2002071486.html.

Lovell, D., Pawlowsky-Glahn, V., Egozcue, J. J., Marguerat, S., and Bähler, J. (2015). Proportionality: A valid alternative to correlation for relative data. *PLoS Comput Biol* 11, e1004075. doi:10.1371/journal.pcbi.1004075.

Martín-Fernández, J., Barceló-Vidal, C., Pawlowsky-Glahn, V., Buccianti, A., Nardi, G., and Potenza, R. (1998). Measures of difference for compositional data and hierarchical clustering methods. in *Proceedings of IAMG*, 526–531.

McMurdie, P. J., and Holmes, S. (2014). Waste not, want not: Why rarefying microbiome data is inadmissible. *PLoS Comput Biol* 10, e1003531. doi:10.1371/journal.pcbi.1003531.

Newey, W. K., and McFadden, D. (1994). "Large sample estimation and hypothesis testing," in *Handbook of econometrics*, eds. R. Engle and D. McFadden (Elsevier Science), 2111—2245.

Pawlowsky-Glahn, V., Egozcue, J. J., and Tolosana-Delgado, R. (2015). *Modeling and analysis of compositional data*. John Wiley & Sons.

Pearson, K. (1897). Mathematical contributions to the theory of evolution. – on a form of spurious correlation which may arise when indices are used in the measurement of organs. *Proceedings of the Royal Society of London* 60, 489–498.

Silverman, J. D., Washburne, A. D., Mukherjee, S., and David, L. A. (2017). A phylogenetic transform enhances analysis of compositional microbiota data. *Elife* 6. doi:10.7554/eLife.21887.

Thorsen, J., Brejnrod, A., Mortensen, M., Rasmussen, M. A., Stokholm, J., Al-Soud, W. A., et al. (2016). Large-scale benchmarking reveals false discoveries and count transformation sensitivity in 16S rRNA gene amplicon data analysis methods used in microbiome studies. *Microbiome* 4, 62. doi:10.1186/s40168-016-0208-8.

Vandesompele, J., De Preter, K., Pattyn, F., Poppe, B., Van Roy, N., De Paepe, A., et al. (2002). Accurate normalization of real-time quantitative rT-pCR data by geometric averaging of multiple internal control genes. *Genome Biol* 3, RESEARCH0034.

Weiss, S., Xu, Z. Z., Peddada, S., Amir, A., Bittinger, K., Gonzalez, A., et al. (2017). Normalization and microbial differential abundance strategies depend upon data

characteristics. *Microbiome* 5, 27. doi:10.1186/s40168-017-0237-y.

Wong, R. G., Wu, J. R., and Gloor, G. B. (2016). Expanding the UniFrac toolbox. *PLoS One* 11, e0161196. doi:10.1371/journal.pone.0161196.
